# Influence of Initial Treatment Modality on Long-Term Control of Chronic Idiopathic Urticaria

**DOI:** 10.1371/journal.pone.0069345

**Published:** 2013-07-23

**Authors:** Sujeong Kim, Seunghee Baek, Bomi Shin, Sun-young Yoon, So Young Park, Taehoon Lee, Yoon Su Lee, Yun-Jeong Bae, Hyouk Soo Kwon, You Sook Cho, Hee-Bom Moon, Tae-Bum Kim

**Affiliations:** 1 Division of Allergy and Clinical Immunology, Department of Internal Medicine, Asan Medical Center, University of Ulsan College of Medicine, Seoul, Korea; 2 Department of Clinical Epidemiology and Biostatistics, Asan Medical Center, Seoul, Korea; 3 Health Screening and Promotion Center, Asan Medical Center, Seoul, Korea; National Institute of Health (NIH), United States of America

## Abstract

**Background:**

Chronic idiopathic urticaria (CIU) is a common cutaneous disorder but the influence of initial treatment modality on long-term control is not known. The aim of this study was to evaluate clinical features, and the influence of initial treatment modality on long-term control.

**Methods and Results:**

641 CIU patients were enrolled from the allergy clinic in a tertiary referral hospital. Disease duration, aggravating factors and treatment modality at each visit were evaluated. Times required to reach a controlled state were analyzed according to initial treatment modality, using Kaplan-Meier survival curves, the Cox proportional-hazards model, and propensity scores. Female to male ratio was 1.7: 1; mean age at onset was 40.5 years. The most common aggravating factors were food (33.5%), stress (31.5%) and fatigue (21.6%). Most patients (82.2%) used H1-antihistamines alone as initial treatment while 17% used a combination treatment with oral corticosteroids. There was no significant difference in the time taken to reach a controlled state between patients treated with single vs multiple H1-antihistamines or between those who received H1-antihistamine monotherapy vs. a combination therapy with oral corticosteroids.

**Conclusion:**

The time required to control CIU is not reduced by use of multiple H1-antihistamines or oral corticosteroids in the initial treatment.

## Introduction

Chronic idiopathic urticaria (CIU) has symptoms that occur daily or almost daily, usually with no obvious stimulus, and that last for more than 6 weeks [1]. At any time, 0.5–1% of the population suffers from CIU (point prevalence) and most patients have an impaired quality of life, comparable with patients who have severe coronary artery disease [2], [3]. The peak age of CIU patients is between 20 and 40 in most studies [4]–[7]. Many CIU patients suffer for longer than a year, but a considerable proportion have symptoms for much longer [2], although results are varied, probably depending on the selection of patients [4], [5], [7]–[11].

Current guidelines from the European Academy of Allergy and Clinical Immunology (EAACI)/Global Allergy and Asthma European Network (GA^2^LEN)/European Dermatology Forum (EDF)/World Allergy Organization (WAO) recommend non-sedating H_1_-antihistamine as the first-line treatment and a four-fold increase in dosage of non-sedating H_1_-antihistamine for patients who do not respond to the standard dose [12]. If that is not effective, second-line treatment including a brief burst of oral corticosteroids is required. However, tertiary referral hospitals may have different patterns of initial treatment when patients exhibit severe symptoms or have not responded to previous treatment. There are few reports on the influence of the initial treatment modality on the long-term control of CIU.

In this study, we examined the clinical characteristics of, and treatment modalities used for, CIU patients in a tertiary hospital in Korea. We also investigated the influence of the initial treatment modality on the long-term control of CIU.

## Materials and Methods

### Study Subjects

The study was conducted in the outpatient allergy clinic of Asan Medical Center, which is a tertiary hospital in Korea. We included patients who first visited our center from September 2007 to March 2011, who had urticaria and/or angioedema without an identifiable exogenous cause for at least 6 weeks or who had clinical features compatible with CIU. We did not include patients who had other forms of chronic urticaria including physical, cholinergic, drug- or food-induced urticaria, or acute urticaria. Nor did we include patients with vasculitis, infectious diseases or dermatologic diseases that might interfere with the evaluation of the symptoms. The medical records of more than 800 patients were reviewed in detail, resulting in a study population of 641.

### Ethics

This study was approved by the Internal Review Board and Ethics Committee of Asan Medical Center and they decided that any informed consent was not needed in this study, because this study was performed by retrospective chart review and the patients' identifications were all deleted.

### Study Design

Demographic and clinical data were collected, including sex, age at onset, body mass index (BMI), duration of symptoms, presence of angioedema, atopic status, aggravating factors, history and response to previous treatment. Laboratory investigations were: complete blood count with differential counts, erythrocyte sedimentary rate (ESR), antinuclear antibodies (ANA), and serum level of complement and total IgE. A skin prick test was performed on patients who reported that food was a possible aggravating factor; they were tested for 32 items to evaluate sensitization to food allergens. The urticaria activity score (UAS) ranged from 0 to 6, depending on the number of wheals (0–3 points) and the intensity of pruritus (0–3 points). In our medical center, the UAS has been included in the electronic medical records since July 2010 according to the current guidelines from EAACI/GA^2^LEN/EDF/WAO [13], [14]. We were therefore able to include the UASs of 106 patients in this study.

The treatment modality and therapeutic response at each visit were also evaluated by reviewing the medical records. We divided the patients into two study groups according to the initial treatment modality. Group I: single H_1_-antihistamine agent *vs.* multiple H_1_-antihistamines; group II: H_1_-antihistamine monotherapy *vs.* combination therapy with oral corticosteroids. The disease was clinically classified as controlled when the symptoms were completely resolved or much improved without changing the treatment (either by increasing the dose or adding another agent) at the next outpatient visit. Finally, we analyzed the time taken to control the disease, according to the initial treatment modality.

### Statistical Analysis

Descriptive statistics (e.g. mean, minimum, maximum, frequency and percentages) were used to describe the demographic data, laboratory findings and aggravating factors. Fisher’s exact tests and χ^2^ tests were used to compare categorical variables, and independent Student’s *t*-tests were used to compare continuous variables. We used a Kaplan-Meier analysis to determine the probability of symptom control at each point in time, and the log-rank test to compare the time to control according to the initial treatment modality. The subjects in each treatment group were matched on a propensity score to minimize the baseline differences in time to control between the groups [15]–[17]. Then we applied the Cox proportional-hazards model to analyze treatment effects in the matched pairs. Variables were the physician, sex, age at onset, concurrent disease, BMI, presence of angioedema, aggravating factors, total IgE, peripheral blood eosinophil count, ESR, and previous treatment history in all cases, and UAS in some cases. Statistical significance was defined as *p*<0.05.

## Results

### Demographic and Clinical Features

Of the 641 patients with CIU, 407 (63.5%) were females, and the female to male ratio was 1.7: 1. The mean age at onset was 40.54 years and the mean duration of symptoms before evaluation was 3.76 years (range, 0.25–650 months). 433 (67.6%) of the 641 patients had urticaria alone; 190 patients (29.6%) had urticaria with angioedema; and 18 patients (2.8%) had only angioedema. 150 patients (23.5%) had a history of atopy. Of these, 99 patients (15.5%) had allergic rhinitis alone, 4.2% had asthma, 1.7% had allergic rhinitis and asthma, and 1.7% had atopic dermatitis. A total of 131 (20.4%) CIU patients underwent diagnostic test for Helicobacter pylori (HP) infection and 83 (63.3%) of them accompanied by HP infection. Some of the subjects had concomitant autoimmune diseases such as Grave’s disease (0.8%), Hashimoto’s thyroiditis (0.2%), rheumatoid arthritis (1.1%), and systemic lupus erythematosus (0.3%). The UAS for assessing CIU activity was measured in 106 patients at the first visit and the mean value was 4.02±1.71 (Table 1).

**Table 1:** Demographic data on the patients with chronic idiopathic urticaria.

Characteristics	N = 641
Sex, M/F, no.	234/407
Age at onset, mean (SD), yr	40.54 (15.11)
BMI, mean (SD), kg/m^2^	23.52 (3.31)
Duration of urticaria at enrollment, mean (SD), yr	3.76 (6.88)
Presence of angioedema, no. (%)	
Urticaria alone	433 (67.6)
Urticaria with angioedema	190 (29.6)
Angioedema alone	18 (2.8)
Personal history of atopy, no. (%)	150/638 (23.5)
Allergic rhinitis	99 (15.5)
Asthma	27 (4.2)
Allergic rhinitis+asthma	11 (1.7)
Atopic dermatitis	11 (1.7)
Concomitant diseases†, no. (%)	
Helicobacter pylori infection*	83 (12.9)
Graves’ disease	5 (0.8)
Hashimoto’s thyroiditis	1 (0.2)
Rheumatoid arthritis	7 (1.1)
Systemic lupus erythematosus	2 (0.3)
Urticaria activity score, mean (SD) (N = 106)	4.02 (1.71)

† Subjects were not accompanied by other autoimmune disorders including celiac disease, type I diabetes mellitus, and Sjögren’s syndrome.

* Helicobacter pylori infection was detected by biopsy urease test including CLO (Campylobacter-Like Organism) test, histology, serologic test to detect H.pylori IgG, or urea breath test. A total of 131(20.4%) CIU patients underwent diagnostic testing for H. pylori.

Abbreviations: BMI, body mass index.

Positive ANA, mostly of low titer, was found in 45 of 403 patients (11.2%). 126 of 438 patients (28.77%) who were tested for ESR had elevated values (>20 mm/h). Serum total IgE level was measured in 458 patients and the mean total IgE level was 278.74±393.54 kU/L (range, 2–3795 kU/L). The mean absolute eosinophil count in peripheral blood was 175.75±181.88 cell/mm^3^ (range, 0–1570.8 cell/mm^3^) and the eosinophil percentage was 2.9±3.18% (Table 2).

**Table 2:** Laboratory data for the patients with chronic idiopathic urticaria.

Laboratory data	
ANA (+), no. (%)	45/403 (11.2) N = 403
PB eosinophil, mean (SD), cell/mm^3^	175.75 (181.88) N = 553
PB eosinophil, % (SD)	2.90 (3.18)
Total IgE, mean (SD), kU/L	278.74 (393.54) N = 458
ESR, mean (SD), mm/hr	16.74 (14.97) N = 438

Abbreviations: ANA, antinuclear antibody; PB, peripheral blood; ESR, erythrocyte sedimentary rate.

**Figure 1:**
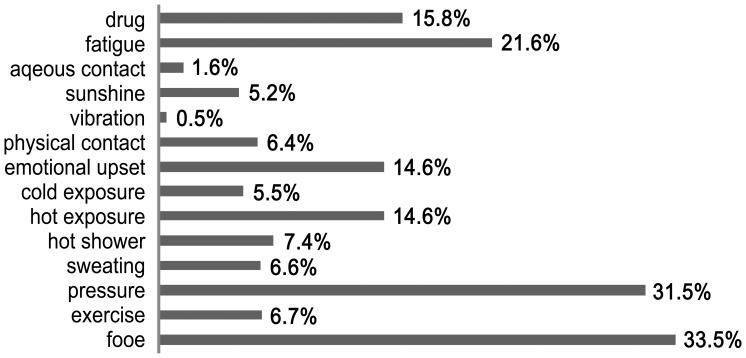
Aggravating factors for chronic idiopathic urticaria based on patient history (N = 639).

Figure 1 shows the possible aggravating factors of CIU based on the histories of the patients. The common aggravating factors were food (33.5%), stress (emotional pressure) (31.5%), and fatigue (21.6%). A skin prick test was performed with food allergens in 103 of the 214 patients who reported food as a possible aggravating factor. 26 patients (25.2%) had a positive response; the common food allergens were shrimp (12.6%), lobster (11.7%), crab (10.7%), and oyster (10.7%). However, no association was found between the symptoms and the results of the skin prick test (data not shown).

### Treatment Modalities

**Figure 2:**
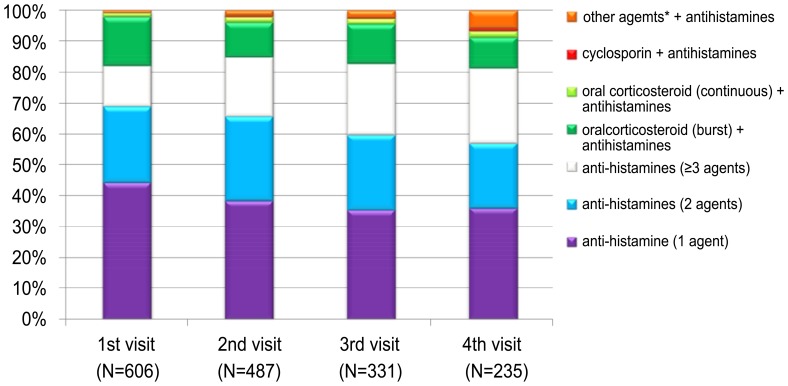
Pattern of prescription at each visit.

As initial treatment, 498 of the 606 patients (82.2%) received H_1_-antihistamines alone and 103 patients (17%) used a combination treatment with oral corticosteroids. However, 96 (15.8%) of the patients treated with oral corticosteroids received corticosteroid therapy for a short time (mean length of time, 5.6 days). Only 7 patients (1.2%) used steroids continuously. Other agents such as a leukotriene antagonist, amitriptyline or dapsone were used in addition to H_1_-antihistamines in 5 patients (0.8%). Levocetirizine, fexofenadine, and ebastine were the most frequently used H_1_-antihistamines (Table S1). There were no significant differences among those different H1-antihistamines in terms of the rate of maintenance of the agent, addition of other agents, and switching to a different H1-antihistamine at the second visit (Table S2). As the number of visits increased, a greater proportion of patients received two or more H_1_-antihistamines, and a smaller proportion received a single agent. The proportion of patients using oral corticosteroids decreased slightly to about 12% after initial treatment. Other agents such as cyclosporine, leukotriene antagonist, amitriptyline and dapsone were added sequentially as patients made subsequent visits (Fig. 2). The time from initial treatment to control CIU according to initial treatment modalities is shown in Table S3.

### Comparison between Single and Multiple H_1_-antihistamines (group I)

In group I, previous treatment history and poor response to previous treatment were more common in patients who received multiple H_1_-antihistamines for the initial treatment, but there was no significant difference in UAS (3.74 *vs.* 4.05, *p = *0.425) (Table 3).

**Table 3:** Baseline characteristics of group I.

Characteristics	Single H_1_-antihistamine (N = 245)	Multiple H_1_-antihistamines(N = 209)	*p* value
Previous treatment history, no. (%)			0.004
No treatment	103/240 (42.9)	59/202 (29.2)	
H_1_-antihistamine	116/240 (48.3)	111/202 (55.0)	
H_1_-antihistamine+steroid	21/240 (8.8)	32/202 (15.8)	
Previous good response, no. (%)			
H_1_-antihistamine	105/115 (91.3)	85/110 (77.3)	0.004
H_1_-antihistamine+steroid	20/21 (95.2)	27/32 (84.4)	0.384
UAS, mean (SD)	(N = 43)	(N = 41)	
	3.74 (1.73)	4.05 (1.75)	0.425

**Figure 3:**
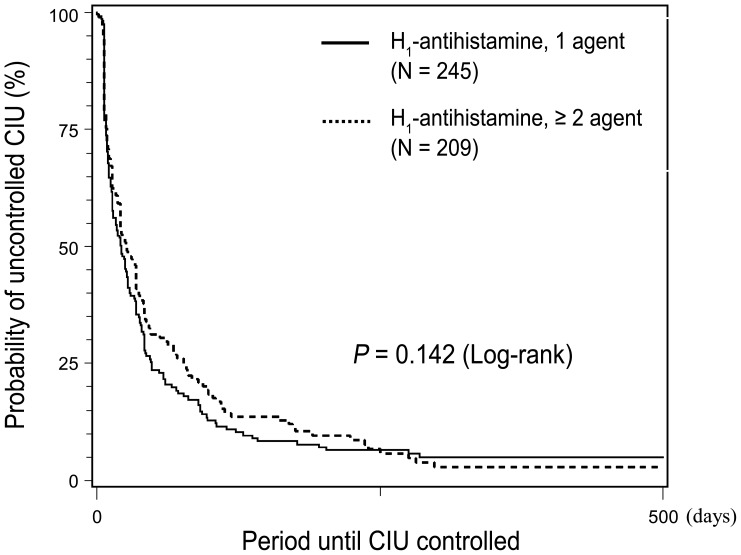
Kaplan-Meier curves showing the relationship between time to control and initial treatment modality (H_1_-antihistamines, single agent *vs.* multiple agents).

In group I, 60.8% of patients who received a single H_1_-antihistamine reached a controlled state one month after initial treatment, compared with 52% of patients who received multiple H_1_-antihistamines. After 3 months, 85% of the single H_1_-antihistamine group and 78.3% of the multiple H_1_-antihistamine group were controlled; after 6 months, these figures were 93.2% and 89.1% respectively. There was no significant difference between the two treatment groups in the time taken to reach control (*p = *0.142) (Fig. 3).

Propensity scores were used to adjust for differences in baseline characteristics between patients treated with single vs. multiple H_1_-antihistamines; 139 pairs of patients were identified. There was still no significant difference in the disease control in the two treatment groups (*p = *0.450). When the UAS values were adjusted, although only 12 matched pairs of patients were identified, the result did not change significantly (*p = *0.660) (Table 4).

**Table 4:** Matched propensity scores from the Cox proportional-hazards model for group I.

Variable		Hazard ratio	95% CI		*p* value
Treatment	Single (reference) *vs.* multiple H1-antihistamines*Single (reference) *vs.* multiple H1-antihistamines#	0.9100.815	0.7130.328	1.1622.024	0.4500.660

* Adjusted variables: physician, sex, age, age at onset, concurrent disease, BMI, presence of angioedema, aggravation factors, total IgE, peripheral blood eosinophil, ESR, previous treatment history.

# Adjusted variables: UAS added to the previous variables.

### Comparison between H1-antihistamine Monotherapy and Combination Therapy with Oral Corticosteroids in Group II

**Figure 4:**
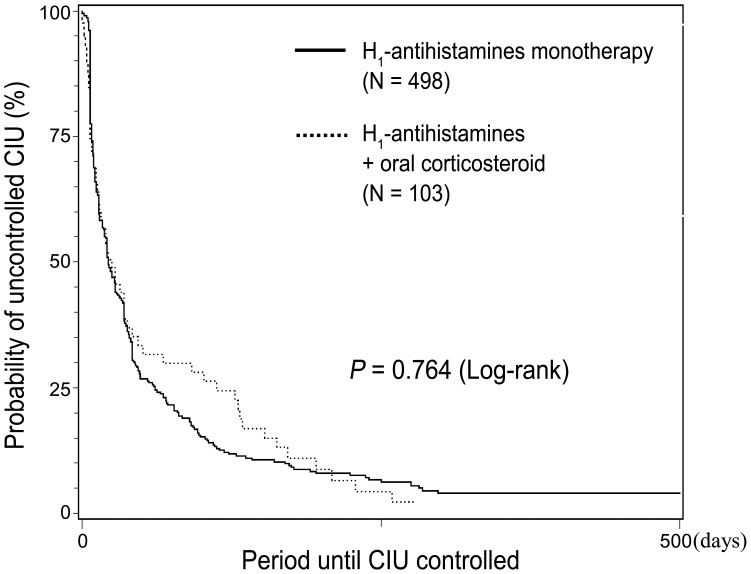
Kaplan-Meier curves showing the relationship between time to control and initial treatment modality (H1-antihistamines monotherapy vs. combination therapy with oral corticosteroids).

Group II was analyzed in the same way as group I. Previous treatment history, poor response to previous treatment and angioedema were more common in patients who received combination therapy with oral corticosteroids, but the UAS was not significantly different (3.89 *vs.* 4.58, *p = *0.191) (Table 5). 56.6% of patients who received H1-antihistamine monotherapy and 54.4% of patients who received combination therapy with oral corticosteroids were controlled within one month after initial treatment. After 3 months, 81.8% of the H1-antihistamine monotherapy group and 71.9% of the combination therapy group were controlled; the figures after 6 months were 91.3% and 89.1% respectively. There was no difference in the time to control between the patients initially treated with antihistamine monotherapy vs. combination therapy with oral corticosteroids (*p = *0.764) (Fig. 4).

**Table 5:** Baseline characteristics of group II.

Characteristics	H_1_-antihistamines monotherapy (N = 498)	H_1_-antihistamines+steroid (N = 103)	*p* value
Presence of angioedema, no. (%)			<0.0001
Urticaria alone	351 (70.5)	57 (55.3)	
Urticaria with angioedema	142 (28.5)	36 (35.0)	
Angioedema alone	5 (1.0)	10 (9.7)	
Previous treatment history, no. (%)			<0.0001
No treatment	162/442 (36.7)	29/95 (30.5)	
H_1_-antihistamine	227/442 (51.4)	32/95 (33.7)	
H_1_-antihistamine+steroid	53/442 (12.0)	34/95 (35.8)	
Previous good response, no. (%)			
H_1_-antihistamine	190/225 (84.4)	16/32 (50.0)	<0.0001
H_1_-antihistamine+steroid	47/53 (88.7)	24/34 (70.6)	0.034
UAS, mean (SD)	(N = 84)	(N = 12)	
	3.89 (1.74)	4.58 (1.38)	0.191

Adjustment for potential confounding factors using propensity scores did not change the result (73 matched pairs, *p = *0.336) (Table 6). However, the UAS value could not be adjusted because too much data was missing in group II.

**Table 6:** Matched propensity scores from the Cox proportional-hazards model for group II.

Variable		Hazard ratio	95% CI		*p* value
Treatment	H_1_-antihistamines (reference) *vs.* H_1_-antihistamines+steroid	1.211	0.820	1.789	0.336

Adjusted variables: physician, sex, age, age at onset, concurrent disease, BMI, presence of angioedema, aggravation factors, total IgE, peripheral blood eosinophil, ESR, previous treatment history.

## Discussion

This is a retrospective observational single-center study of the patients with CIU in Korea. The predominance of women in our study corresponds with that in US, European and Asian studies [5], [7], [18]–[20]. The mean age at the time of enrollment was 44.2 years, which is higher than that of other retrospective studies in a single center [5], [18], [19]. The mean duration of urticarial symptoms at enrollment was 3.76 years, compared with 2.87 years in other study [18]. The association of angioedema with CIU was lower (29.6%) than in other studies, in which it was 33–67% of the study population [2]. In the present study, the frequency of personal histories of atopy was 23.5% including allergic rhinitis (15.5%) and asthma (4.2%). In Korea, Choi *et al.* reported in 1983 that 8.8% of 147 chronic urticaria patients who visited a tertiary hospital had a history of atopic disease [21]. In 2000, Lee *et al.* reported that 21.2% of 113 patients with chronic urticaria had atopic diathesis, and that 18.6% of these had allergic rhinitis and 1.8% asthma [22]. Although CIU was not distinguished from other forms of non-acute urticaria in these two studies, the data imply that atopy has become more prevalent in patients with CIU over the past 20 years.

Five hundreds of 639 patients with CIU (78.2%) had one or more aggravating factors. This finding is consistent with the result of a previous study in Korea showing that 70.1% of patients reported aggravation of their symptoms by one or more eliciting factors, but the major factors in the two studies are different. In the previous study conducted in another tertiary hospital in Korea, the common aggravating factors were stress or nervousness (40.1%), exposure to heat (33.3%), exposure to cold (18.4%), and sweating (15%) [21]. In contrast, in the present study food, emotional pressure and fatigue were the main aggravating factors, with exposure to heat and cold as the fifth and tenth causes, respectively. Stress and related conditions such as fatigue and emotional upset seemed to aggravate CIU in both studies. Some clinical data have suggested stress as an eliciting and/or exacerbating factor of CIU. Chung *et al.* found that patients with CIU were 1.89 times more likely to have a current diagnosis of posttraumatic stress disorder (PTSD) than the control group [23]. Another study also shows that CIU patients had higher levels of life event stress and perceived stress than a control group [24].

Symptomatic therapy with a non-sedating H_1_-antihistamine is the main treatment in most patients. However, treatment with licensed doses of H_1_-antihistamines relieves the symptoms in fewer than 50% of patients [25]–[30]. In the present study, a single non-sedating H_1_-antihistamine was used in 44.2% of the study population as the initial treatment, 38% of the population used two or more H_1_-antihistamine agents, and 17% of the patients used oral corticosteroids in bursts or continuously. A study conducted at Beth Israel Deaconess Medical Center reported that H_1_-antihistamine monotherapy was administered to 37% of CIU patients and corticosteroid or cyclosporine was used in 28% of the study population. These data show that corticosteroid or cyclosporine is more likely to be used in the first 6 months or after 5 years of the disease (29% and 30% of patients, respectively) [18].

A combination of more H_1_-antihistamine agents or a short course of oral corticosteroids at the start of treatment is often prescribed in tertiary hospitals because patients require faster relief of symptoms. Corticosteroids may help reduce the duration of acute urticaria [31]. However, there is no clear evidence that they are effective for the long-term control of CIU. According to our results, the time to reach stable, long-term control was not reduced by adding different H_1_-antihistamines or oral corticosteroids to a single H_1_-antihistamine in the initial treatment. There may be some advantages in use single H1-antihistamine modality in the initial treatment of CIU. First, this strategy could reduce medication cost. Second, this could prevent the patients from undergoing potential adverse effects by taking high doses of antihistamines. In addition, overuse of oral corticosteroids in the initial treatment can be reduced through our study, because many practitioners tend to use oral corticosteroids for the purpose of quick relief of symptoms. However, we don’t mean that oral corticosteroids should not be used even in cases of severe or exacerbated CIU. We tried to investigate whether it is reasonable or not to use multi-modality drugs as initial treatment of CIU in aspect of long-term prognosis. And, our findings cannot be applied for use of other agents such as cyclosporine, leukotriene antagonist, amitriptyline, or dapsone.

To overcome the limitations of a retrospective observational study, and to reduce the impact of treatment-selection bias, we applied propensity scores. However, we still found that control of the disease was not affected by initial combination therapy with additional H_1_-antihistamine or oral corticosteroids. To our knowledge, this is the first study concerning the influence of initial treatment modality on the long-term control of CIU.

One limitation of the study was the lack of UASs for assessing CIU activity in patients and monitoring their response to treatment. Also, because we had to evaluate the patients during clinic visits, we could not detect changes in the disease between visits.

In conclusion, this study provides insight into the clinical features of CIU patients in a tertiary referral hospital in Korea. Our data suggest that physicians should not routinely prescribe multiple H_1_-antihistamines, or oral corticosteroids, in patients who have CIU, unless they present with very severe symptoms. Additional well-designed prospective randomized control studies are needed to provide robust evidence of the therapeutic efficacy of each treatment modality, and this may contribute to the development of practical treatment strategies for CIU.

## Supporting Information

Table S1 **Non-sedating H1-antihistamines used as initial treatment.**
(DOCX)

Table S2 **The rate of maintenance, addition of other agents, and switching to a different H1-antihistamine in the most commonly used H1-antihistamines.**
(DOCX)

Table S3 **The time from initial treatment to control chronic idiopathic urticaria according to initial treatment modalities.**
(DOCX)
