# Supplementary material for: Influence of Initial Treatment Modality on Long-Term Control of Chronic Idiopathic Urticaria
Source: PLoS One. 2013 Jul 23;8(7):e69345. doi: 10.1371/journal.pone.0069345 (PMC3720657; doi:10.1371/journal.pone.0069345)
Supplement: Table S1 — Non-sedating H1-antihistamines used as initial treatment. (DOCX) [file pone.0069345.s001.docx]

Table S1. Non-sedating H_1_-antihistamines used as initial treatment

| **Prescribed H_1_-antihistamines** | **N. of patients*** | **%** |
| --- | --- | --- |
| Levocetirizine | 140 | 55.5 |
| Fexofenadine | 69 | 27.4 |
| Ebastine | 31 | 12.3 |
| Loratadine | 8 | 3.2 |
| Bepotastine | 2 | 0.8 |
| Mequitazine | 2 | 0.8 |

* The number of patients prescribed each single agent of non-sedating H_1_-antihistamine for initial treatment
