# Supplementary material for: Influence of Initial Treatment Modality on Long-Term Control of Chronic Idiopathic Urticaria
Source: PLoS One. 2013 Jul 23;8(7):e69345. doi: 10.1371/journal.pone.0069345 (PMC3720657; doi:10.1371/journal.pone.0069345)
Supplement: Table S2 — The rate of maintenance, addition of other agents, and switching to a different H1-antihistamine in the most commonly used H1-antihistamines. (DOCX) [file pone.0069345.s002.docx]

Table S2. The rate of maintenance, addition of other agents, and switching to a different H1-antihistamine in the most commonly used H_1_-antihistamines

|  | **Visit 1** | **Visit 2** | |
| --- | --- | --- | --- |
| Levocetirizine | N = 140 |  | N = 96 |
|  |  | Maintain | 60 (62.5%) |
|  |  | Add* | 24 (25.0%) |
|  |  | Switch^#^ | 12 (12.5%) |
| Fexofenadine | N = 69 |  | N = 55 |
|  |  | Maintain | 34 (61.8%) |
|  |  | Add | 19 (34.5%) |
|  |  | Switch | 2 (3.6%) |
| Ebastine | N = 31 |  | N = 25 |
|  |  | Maintain | 18 (72.0%) |
|  |  | Add | 5 (20.0%) |
|  |  | Switch | 2 (8.0%) |

*Add other agents including another H_1_-antihistamines, oral corticosteroids, cyclosporine, leukotriene antagonist, amitriptyline or dapsone.

^#^Switch to a different H_1_-antihistamine
