# Supplementary material for: Influence of Initial Treatment Modality on Long-Term Control of Chronic Idiopathic Urticaria
Source: PLoS One. 2013 Jul 23;8(7):e69345. doi: 10.1371/journal.pone.0069345 (PMC3720657; doi:10.1371/journal.pone.0069345)
Supplement: Table S3 — The time from initial treatment to control chronic idiopathic urticaria according to initial treatment modalities. (DOCX) [file pone.0069345.s003.docx]

Table S3. The time from initial treatment to control chronic idiopathic urticaria according to initial treatment modalities

| **Treatment modalities** | **Total N.** | **N. of controlled cases (%)** | **N. of censored cases* (%)** | **Time to control, days,**  **mean (95% CI)** | **Time to control, days,**  **median (IQR)** |
| --- | --- | --- | --- | --- | --- |
| Anti-histamine (1 agent) | 268 | 182 (67.9) | 86 (32.1) | 63.1 (44.5-81.6) | 22 (8-49) |
| Anti-histamines (≥ 2 agents) | 230 | 163 (70.9) | 67 (29.1) | 68.9 (50.5-87.4) | 27 (9-77) |
| Oral corticosteroid (burst)  + antihistamines | 96 | 70 (72.9) | 26 (27.1) | 75.9 (45.7-106.1) | 22 (7-113) |
| Oral corticosteroid (continuous) + antihistamines | 7 | 6 (85.7) | 1 (14.3) | 89.1 (3.4-174.9) | 35 (10-153) |
| Other agents^†^ + antihistamines | 5 | 4 (80) | 1 (20) | 56.8 (13.3-100.3) | 36 (30-61) |

*Censored cases because of loss to follow-up or end-of-observation for an endpoint of control

^†^ Leukotriene receptor antagonist, amitriptyline, or dapsone

Abbreviations: 95% CI, 95% confidence interval; IQR, interquartile range
